# S10-1: Using open GIS data to identify bikeable neighbourhoods for adolescents across Germany: Results of the MoMo-Study

**DOI:** 10.1093/eurpub/ckae114.241

**Published:** 2024-09-26

**Authors:** Leon Klos, Rebecca Pedrick-Case, Richard Fry, Hagen Wäsche, Claudia Niessner, Alexander Woll

**Affiliations:** Institute of Sports and Sports Science, Karlsruhe Institute of Technology, Germany; Department of Health Data Science, Swansea University, UK; Department of Health Data Science, Swansea University, UK; Institute of Sports and Sports Science, Karlsruhe Institute of Technology, Germany; Institute of Sports and Sports Science, Karlsruhe Institute of Technology, Germany; Institute of Sports and Sports Science, Karlsruhe Institute of Technology, Germany

## Abstract

**Purpose:**

Bikeability is an emerging concept that quantifies the perceived comfort and convenience of accessing important destinations by bike. Existing bikeability indices are limited to adults, single, large cities and use restricted GIS sources. The aims of this study are to (1) identify environment characteristics related for a bikeable neighbourhood that can be analysed using open GIS data and (2) find associations between bikeability measures and cycling behaviour in adolescents across Germany.

**Methods:**

Street network data, and location data on schools, shops, food establishments, parks and outdoor pitches were downloaded from OpenStreetMap, topography data was taken from the European Digital Elevation Model and population data from the German census. A routing algorithm was used to model the access to the different destinations and to measure the length of streets with slow traffic and separated cycling infrastructure within 4-20 minute bike rides. Data from the MoMo study wave 3 (1253 adolescents aged 11-17 years, 50.1% female) were used to assess the relationship between the GIS measures and cycling to school and non-motorized vehicle use (e. g. cycling, longboarding).

**Results:**

Preliminary results indicate that access to all types of locations is positively correlated with cycling to school, strongest within the 12 and 16 minute bike ride buffer. Smaller associations were found between accessibility and non-motorized vehicle use. The length of dedicated cycling infrastructure and residential streets within an 8-16 minute bike ride are related to cycling to school. Results on separated cycling infrastructure along main streets are inconsistent.

**Conclusions:**

We demonstrated that openly available GIS data can be used to quantify bikeable neighbourhoods for adolescents across Germany. Safe cycling infrastructure, roads with low speed limits, and access to different places of interest are crucial to promote cycling in adolescents, strengthening the evidence for the European Declaration on Cycling.

**Support/Funding Source:**

This work has been developed within the Motorik-Modul Longitudinal Study (MoMo) (2009 – 2022) MoMo is funded by the Federal Ministry of Education and Research (funding reference number: 01ER1503).

